# The effects of isoflavone supplementation plus combined exercise on salivary markers of oxidative stress in postmenopausal women

**DOI:** 10.3164/jcbn.19-44

**Published:** 2019-10-31

**Authors:** Ana Luiza Amaral, Anne M. Mendonça, Jéssica S. Giolo, Juliene G. Costa, Igor M. Mariano, Tállita C. F. de Souza, Jaqueline Pontes Batista, Mateus L. Rodrigues, Adriele V. de Souza, Douglas C. Caixeta, Leonardo G. Peixoto, Erick P. de Oliveira, Foued S. Espindola, Guilherme M. Puga

**Affiliations:** 1 Laboratory of Cardiorespiratory and Metabolic Physiology, Federal University of Uberlândia,Uberlândia-MG 38400-678, Brazil; 2 Department of Food and Human Nutritional Sciences, University of Manitoba, Winnipeg-MB, R3T 2N2, Canada; 3 Canadian Centre for Agri-Food Research in Health and Medicine, Winnipeg-MB, R2H 2A6, Canada; 4 Laboratory of Biochemistry and Molecular Biology, Institute of Biotechnology, Federal University of Uberlândia, Uberlândia-MG 38400-902, Brazil; 5 School of Medicine, Federal University of Uberlândia, Uberlândia-MG 38400-902, Brazil

**Keywords:** aerobic and resistance exercises, soy protein, menopause, anti-oxidant system

## Abstract

This study tested the effect of isoflavone supplementation in addition to combined exercise training in salivary oxidative stress markers in non-obese postmenopausal women. Thirty-two postmenopausal women without hormone therapy were randomly assigned to exercise + placebo (*n* = 15) or exercise + isoflavone supplementation (*n* = 17) groups. They performed 30 sessions of combined exercises (aerobic plus resistance) over ten weeks and consumed 100 mg of isoflavone supplementation or placebo. Saliva samples were collected after an overnight fast. Superoxide dismutase, total antioxidant capacity, thiobarbituric acid reactive substances, catalase, total protein and nitrite were determined before and after ten weeks of the intervention. The ANOVA two-way analysis of variance was applied with α of 5%. Both groups increase (*p*<0.05) superoxide dismutase activity and decrease catalase levels. There was interaction (time × group) in both nitrite and thiobarbituric acid reactive substances results, with increase (*p*<0.05) in placebo group and decrease (*p*<0.05) in isoflavone group. No difference was found for total antioxidant capacity or total protein. The combination of isoflavone supplementation and exercise training can promote an antioxidant effect through reduction of lipid peroxidation and concentrations of salivary nitrite.

## Introduction

Women in post menopause are predisposed to the development of cardiovascular diseases,^(1)^ and one possible reason to that is an increase in oxidative stress during this life period,^(2)^ leading to endothelial cells dysfunction.^(3)^ Endothelial cells are responsible for biological functions, mainly in cardiovascular and endocrine systems. Further, a prooxidant-antioxidant balance is necessary in order to have these cells functioning properly.^(3,4)^ Oxidative stress is an imbalance of this system to prooxidant molecules, thus, generating more reactive oxygen species (ROS), which are involved in several cardiovascular diseases, inflammation, cancer and aging.^(3–5)^ Clinically, there are methods to evaluate oxidative stress markers, such as the enzymes catalase and superoxide dismutase (SOD),^(6)^ and also nitrite,^(7)^ an important nitric oxide (NO) precursor and a key to endothelium vasoprotection.^(8)^

Saliva is an important factor in oral health maintenance of individuals.^(9)^ Salivary analysis for oxidative stress markers is an effective and non-invasive method,^(10)^ however research about salivary oxidative markers are still lacking. An imbalance of ROS and consequent changes in oxidative stress salivary markers are associated with not only oral diseases, such as dental caries,^(9,10)^ and periodontal diseases,^(10,11)^ but also with other systemic diseases, such as type II diabetes,^(11,12)^ and obesity and it also increases with aging process.^(13,14)^ Importantly, studies have shown that salivary free radical-scavenging activity was affected by physical and mental activities.^(15)^ Furthermore, some studies suggest that saliva may reflect the current physiological condition of the body.^(16,17)^

The maintenance of a prooxidant-antioxidant balance is important to keep healthy conditions to physiological activities, and there are reports that physical exercise leads to positive responses to this balance.^(7,18,19)^ Regular exercise training has been demonstrated to improve antioxidant defense,^(18,19)^ and to increase nitrite concentrations.^(20)^ Acute exercise results in an increased production of ROS,^(21)^ however, chronic exercise promotes an adaptation to the antioxidant defense system, leading to adequate responses to the higher ROS generation.^(19,22)^ Moderate intensity aerobic training promotes mechanisms for this adaptation, such as an increased production of endothelium vasoactive substances.^(23)^ Further, the combination of aerobic with resistance exercise has been demonstrated to effectively diminish ROS production, and enhance the enzymatic antioxidant defense.^(24)^

Associated with physical exercise, postmenopausal women often use alternative treatments to reduce climacteric symptoms, mainly supplements structurally similar to estrogen, such as isoflavones, a phytoestrogen derived from soy which can change the oxidative balance.^(25)^ Studies have controversial data regarding oxidative stress markers and isoflavone supplementation (ISO). In some studies, there was a decrease in ROS production, and an increase in SOD in postmenopausal women,^(26,27)^ other showed that genistein, a soy isoflavone, or the soy isoflavone from hypocotyl could decrease oxidative stress via lowering lipid peroxidation, and improving antioxidant enzymes.^(28,29)^ Besides, other studies find that a high isoflavone consumption, as well as exercise, is effective in increasing nitrite concentrations and improving endothelial function.^(30,31)^ However, there are also studies that did not report any effect in oxidative markers with ISO.^(32)^

Therefore, the objective of this study was to verify if there is an additional effect of ISO associated with combined aerobic and resistance exercise training, in salivary oxidative stress markers in postmenopausal women. Our hypothesis was that ISO together with combined exercise performance would improve oxidative stress markers.

## Materials and Methods

This study was a randomized, double-blind, parallel prospective clinical trial, carried out at the Laboratory of Cardiorespiratory and Metabolic Physiology of the Physical Education Department at the Federal University of Uberlândia, Uberlândia MG, Brazil. The study was approved by the local Ethics Committee for human studies (CAAE: 40622414.9.0000.5152). All participants agreed and signed an informed consent form prior to admission to the study, and experiments were performed in accordance to the World Medical Association Declaration of Helsinki. This research was registered at clinicaltrials.gov number NCT03008785.

### Participants

**Fig. 1:**
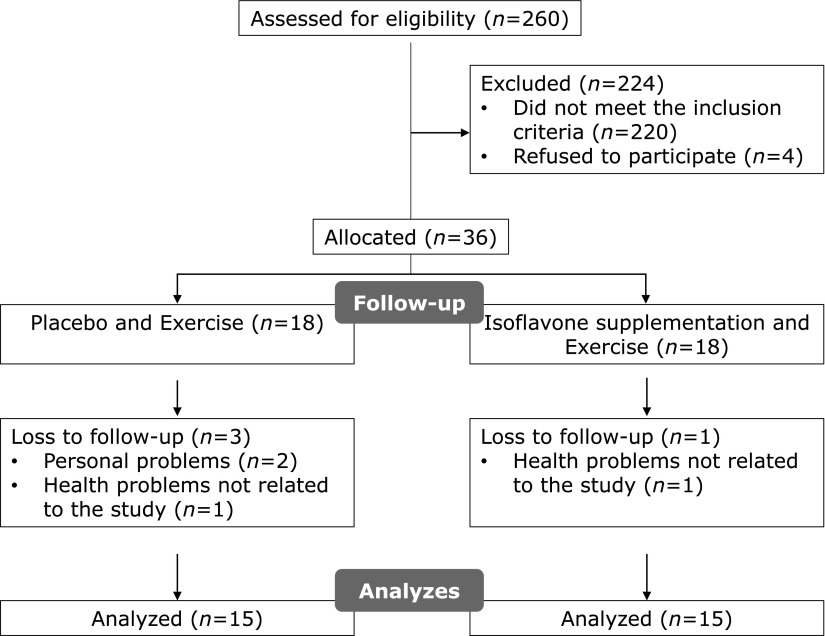
Follow-up flowchart.

Postmenopausal women, aged between 50 and 70 years old, were recruited by traditional media (TV, radio and posters). The inclusion criteria were: a body mass index (BMI) ≤30 kg/m^2^, amenorrhea for at least 12 months, ability to participate in treadmill and resistance exercises, no hormone therapy or supplementation of soy derived products, no use of drugs that alter lipid profile, and no history of high blood pressure, diabetes or cardiovascular disease. Fig. 1 presents the flowchart with the distribution of the participants in the study.

All participants who met the inclusion criteria were randomly allocated, through electronic lottery, to two different groups: exercise training and ISO or exercise training and placebo. Saliva samples of all participants were collected before and after 10 weeks of placebo or ISO, and combined aerobic and resistance exercise training. Participants also went through anthropometric measurements, a test for aerobic capacity and maximum strength (1-RM) and were familiarized with the ergometers and exercises of the trial prior to start.

### Supplementation

ISO group received one capsule per day of 100 mg of isoflavones containing 3.3 mg of genistein, 93.5 mg of dadzein and 3.2 mg of glycitein derived from soybean (Xi’An Green-Life Natural Products, Xian, Shaanxi, China). These amounts correspond to approximately 37.58 g of soy.^(33)^ Placebo group received one capsule per day containing 100 mg of cornstarch. The capsules for both groups were identical in appearance, smell, and flavor.

### Dietary assessment

Food intake was assessed by trained nutritionists that instructed all participants to maintain their regular diet throughout the study. Three dietary recalls (two weekdays and one weekend day) were gathered on non-consecutive days at baseline and at the end of study. Dietary data was analyzed by the United States Department of Agriculture (USDA) food composition table and by the software by Dietpro 5.7i (Viçosa, Brazil) and there was no difference in dietary pattern between groups during the intervention (data not shown).

### Body composition

Height and weight were measured using a standard stadiometer Sanny (São Paulo, Brazil) and an electronic scale Micheletti (São Paulo, Brazil), respectively. BMI was calculated and rated according to the World Health Organization (WHO).^(34)^ To assess total fat mass and fat-free mass participants went through a bioimpedance test (Biodynamics model 450c; Biodynamics, Shoreline, WA). The test was performed in the morning after at least eight hours of fasting, and hydration was controlled as previously reported.^(5)^

### Exercise training

Exercise training was performed as previously described.^(32)^ Briefly, the exercise training consisted of 30 sessions of combined aerobic and resistance exercises during the same session. Exercise sessions took place three times per week on non-consecutive days, over 10 weeks. All sessions included a warm-up on a treadmill for 5 min, aerobic exercise on a treadmill for 20 min, resistance exercises for 20 min, and cool down exercises for 5 min, with a total of 50 min for each session. Aerobic and resistance exercise order was changed in every training session. All women went through a familiarization session with the treadmill and resistance exercises before the intervention start.

### Saliva samples collection and analysis

Saliva samples were collected after an overnight fast, five days before, and 72 h after, the last exercise training session to eliminate possible acute effects of exercise. All volunteers were instructed not to consume alcoholic beverages or caffeine, and not to practice intense physical activities 24 h prior to sample collection. After a 30 s mouthwash with distilled water, unstimulated saliva was collected into graduated Falcon tubes by the spitting method for 2 min.^(35)^ Samples were centrifuged at 3,500 rpm and 4°C for 15 min, transferred to microtubes of 1.5 ml and stored at −80°C. All analyses were performed using an automated system (Cobas Mira, Roche Instruments Inc. Bellport, NY), using commercial kits (Labtest, Minas Gerais, Brazil).

The total antioxidant capacity in saliva was evaluated by the ferric reducing ability potential (FRAP) methodology and calculated from the standard trolox curve, as described by Justino *et al.*^(36)^ The activity of the SOD was determined based on the auto-oxidation capacity of pyrogallol.^(36)^ Lipid peroxidation levels were analyzed by the thiobarbituric acid reactive substances (TBARS) method, using a curve of 1,1,3,3-tetramethoxypropane (TMP) as the standard.^(36)^ The salivary nitrite, catalase and total protein (TP) were measured as described by Giolo et al.^(32)^

### Statistics

Results are reported as means ± SE. Data distribution was analyzed using the Shapiro-Wilk test. Sample size was calculated using data from G*Power 3.0.10, using an α value of 0.05, a β value of 0.15 and a power analysis of 85% with a final number of volunteers of 26 women. The two-way Analysis of Variance (ANOVA) for repeated measures was used to analyze the time (pre and post), group (placebo and ISO) and group vs time interactions with a Bonferroni post hoc test, when appropriate. A *p* value of <0.05 was set for statistical significance, and all statistical analyses were performed using SPSS software ver. 20.0 (IBM, NewYork, NY).

## Results

ISO group had mean age of 56 ± 5.40 years, body mass of 65.35 ± 8.73 kg, height of 1.57 ± 0.04 m, BMI 26.29 ± 3.38 kg/m^2^, and abdominal circumference of 93.15 ± 9.72 cm. The placebo group had a mean age of 52.67 ± 4.90 years, body mass of 64.67 ± 8.60 kg, height of 1.56 ± 0.06 m, BMI of 26.52 ± 3.14 kg/m^2^, and abdominal circumference of 92.80 ± 7.57 cm.

**Fig. 2:**
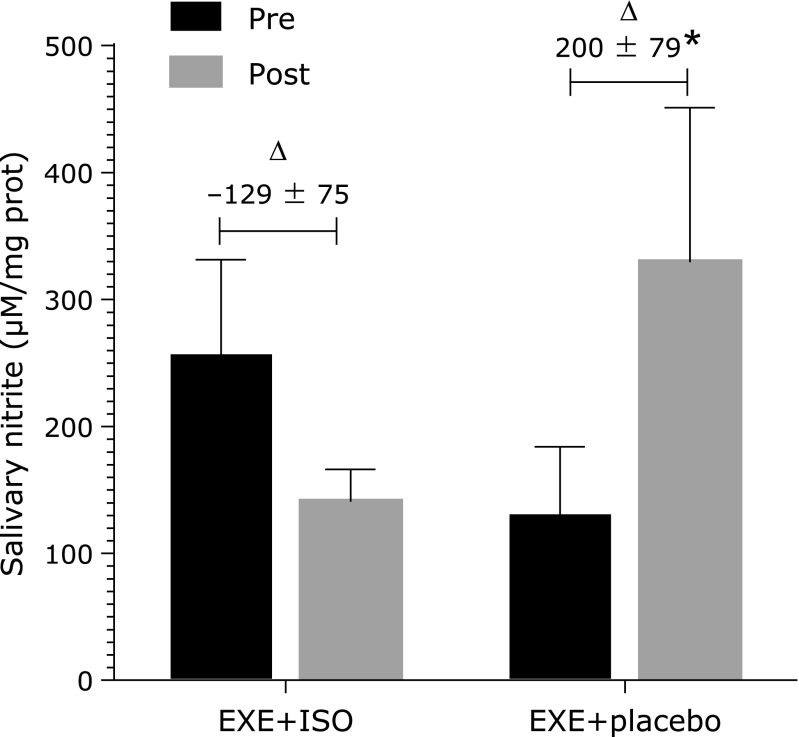
Salivary nitrite of postmenopausal women in placebo and ISO groups, in pre- and post-intervention moments. *p = 0.007.

Oxidative stress markers in saliva are displaced in Fig. 2 and Table 1. There were significant time effects in catalase (decreases in both groups) and SOD (increase in both groups) concentrations, whereas no interaction, time or group effect was found for FRAP or TP. There was an interaction for nitrite concentrations in saliva (*p* = 0.007), in which the concentrations for placebo group increased and ISO group decreased after the intervention (Fig. 2). There was also an interaction for TBARS concentrations (*p* = 0.035) in which this marker was slightly lower in ISO group and slightly increased in placebo group after intervention (Table 1). Catalase concentration was slightly lower after intervention in both groups with a significant effect of time (*p* = 0.035). SOD concentration was increased in both groups after the exercise protocol with a significant effect of time (*p* = 0.001).

## Discussion

To the best of our knowledge this is the first-time salivary oxidative markers of postmenopausal women have been evaluated before and after 10 weeks of ISO with combined aerobic and resistance exercise. This study demonstrates an effect of the association of isoflavone with exercise for nitrite and TBARS concentrations in saliva, indicating lower production of nitrate with ISO, and a slightly lower production of lipid peroxides by the TBARS evaluation. Combined exercise had a positive effect on SOD, which had its concentration increased, and a negative effect on catalase, that was slightly lower after the exercise protocol in both groups.

Besides that, during physical exercise, the high shear stress caused by blood in vessels can lead to an increase in the release of a potent vasodilator named nitric oxide (NO), that improves endothelial function.^(20)^ However, NO can react rapidly with the anion superoxide resulting in peroxinitrite, a potent oxidant related to mitochondrial damage and increase of oxidative stress.^(8)^ In this study, there was a chronic increase in salivary nitrite concentrations (i.e., NO precursor) in the placebo group, which can be explained by the mechanism of shear stress stimulated by exercise. Previous studies have shown an increase of salivary nitrite in active man in up to one hour after aerobic exercise,^(37)^ and after combined exercise in patients with peripheral arterial disease.^(38)^ Besides, the nitrite/nitrate concentrations were also higher after three months of an exercise protocol in healthy postmenopausal women.^(39)^ Interestingly, in ISO group nitrite concentrations were lower after intervention. ISO can also increase serum nitrite/nitrate, and improve endothelial function, as well as exercise, in postmenopausal women.^(30,31)^ Although some studies show that both isoflavone consumption, and exercise alone can increase nitrite concentrations, our results indicate that when associated, isoflavone does not seem to have any additional benefits to this variable.

Therefore, there are other mechanisms that may explain nitrite reduction in ISO group. A study showed that genistein and dadzein can promote an antioxidant mechanism through NO or peroxinitrite clearance consequently reducing deoxyribonucleic acid (DNA) damage.^(40)^ There was also a study showing that isoflavones can attenuate excessive NO generation *in vitro*.^(41)^ In our study, it is possible that ISO blocked the increase in salivary nitrite induced by exercise training and lowered its concentration in order to prevent peroxinitrite production and an increase in oxidative stress in postmenopausal women.

Besides decreasing nitrite, our study shows that ISO also slightly decreased TBARS production in postmenopausal women engaged in a combined exercise program. One of the theories for this event is the increase in peroxynitrite, a free radical that can induce the oxidation of low density lipoprotein (LDL) and increase the risk of developing atherosclerosis.^(42)^ Accordingly, studies have shown decreased TBARS concentrations with ISO in postmenopausal women and in animal models.^(43,44)^ Isoflavones, including dadzein and genistein, are able to inhibit TBARS formation, demonstrating an antioxidant effect.^(45,46)^ Some authors showed that chronic soy ISO even from hypocotyl can decrease lipid perioxidation and some pro inflammatory cytokines markers.^(29)^ The efficiency of TBARS inhibition may depend on the individual isoflavones concentration in the extract, with some compounds having low or moderate efficiency to inhibit TBARS,^(46)^ which may explain the slight interaction observed in this study (*p* = 0.037), and also why other studies found no changes in lipid peroxidation markers with ISO.^(32,33)^

One of the main benefits from exercise training is related to improvements in antioxidant defense. In this way, we found an increase in SOD in both groups after combined exercise training indicating that this type of exercise training, independently of ISO, can improve the antioxidant activity of this enzyme in saliva. This data was already expected, since exercise training leads to an adaptation of the antioxidant system by increasing prooxidants cells in each exercise session.^(21,47)^ Therefore, in the long-term, exercise training stimulates the antioxidant defense system to attenuate the acute effect generated by exercise.^(23,47)^

The limitations of this study must be taken into consideration. The study was conducted with healthy, non-obese women; therefore, the results might not be applicable to groups receiving higher potency medical treatment, or for longer than 10 weeks. It is important to note that this result is applicable only for ISO and may not be extrapolated to isoflavone consumption from natural and regular food and nor in different amounts of isoflavone and concentrations of its compounds. Other limitations were the absence of an evaluation of isoflavone levels in the urine to detect how much soy protein was actually absorbed in the study population. More studies are needed with larger numbers of participants and with patients with dyslipidemia, obesity or with high oxidative stress levels to investigate the effects of these interventions.

ISO associated with a combined exercise program can promote an antioxidant effect through reduction of lipid peroxidation and concentrations of salivary nitrite. Furthermore, combined exercise training alone can increase the antioxidant activity of the enzyme SOD after 10 weeks of intervention. Differences in salivary FRAP, catalase and TP were not detected after the intervention period.

## Author Contributions

ALA and JSG participated in the data collection and analysis, performed statistical analysis, and wrote the manuscript; AMM contributed with English review and wrote the manuscript; IMM participated in the data collection and analysis, performed statistical analysis and contributed with the revision of the manuscript; JGC, TCFS and JPB participated in the data collection, and contributed with the revision of the manuscript; MLR and AVS participated in the data analysis, performed statistical analysis and contributed with the revision of the manuscript; DCC and LGP participated in the saliva data analysis, performed statistical analysis and contributed with the revision of the manuscript; EPdO and FSE participated in the study design, elaboration of the discussion and with the revision of the manuscript; GMP contributed with the study design, data collection and analysis, statistical analysis, and with the manuscript elaboration and review. All authors read and approved the final manuscript.

## Figures and Tables

**Table 1:** Oxidative stress markers in saliva of postmenopausal women

	Pre	Post	Δ	*p*	*p*	*p*	Effect size
	Mean ± SE	Mean ± SE		(Groups)	(Moments)	(Groups * Moments)	
CATALASE (U/mg prot)							
ISO	21.32 ± 2.30	16.02 ± 2.04	−5.29 ± 1.48	0.460	0.035	0.708	0.008
placebo	17.59 ± 2.45	16.28 ± 2.37	−1.25 ± 1.46				
FRAP (µmol/L eq. Trolox)							
ISO	42.28 ± 3.56	38.49 ± 4.80	−3.57 ± 3.90	0.970	0.064	0.120	0.291
placebo	50.29 ± 8.01	59.09 ± 11.61	8.04 ± 9.72				
SOD (U/mg prot)							
ISO	5.36 ± 0.63	8.66 ± 0.66	2.91 ± 0.73	0.669	0.001	0.790	0.154
placebo	3.91 ± 0.71	11.16 ± 2.22	6.74 ± 2.07				
TBARS (µmol/mg prot)							
ISO	4.93 ± 0.24	4.52 ± 0.18	−0.41 ± 0.17	0.698	0.735	0.037	0.21
placebo	5.02 ± 0.29	5.57 ± 0.44	0.26 ± 0.67				
TP (µg/µl)							
ISO	0.64 ± 0.05	0.65 ± 0.04	0.01 ± 0.03	0.999	0.133	0.221	0.078
placebo	0.69 ± 0.04	0.76 ± 0.06	0.07 ± 0.04				

Interaction: groups vs time interaction; ISO, isoflavone supplementation; FRAP, plasma ferric reduction capacity; SOD, superoxide dismutase; TBARS, thiobarbituric acid reactive substances; TP, total protein

## Abbreviations

ANOVA: analysis of variance
BMI: body mass index
DNA: deoxyribonucleic acid
FRAP: total antioxidant capacity
ISO: isoflavone supplementation
LDL: low density lipoprotein
NO: nitric oxide
1-RM: maximum strength
ROS: reactive oxygen species
SOD: superoxide dismutase
TBARS: thiobarbituric acid reactive substances
TMP: 1,1,3,3-tetramethoxypropane
TP: total protein
USDA: United States Department of Agriculture
WHO: World Health Organization
